# Acorn Availability Reduces Agricultural Damage by Ungulates

**DOI:** 10.1002/ece3.73974

**Published:** 2026-07-27

**Authors:** Maria A. Bogdańska, Valentin Journé, Michał Bogdziewicz

**Affiliations:** ^1^ Forest Biology Center, Faculty of Biology, Institute of Environmental Biology Adam Mickiewicz University Poznań Poland; ^2^ Department of Biology, Faculty of Science Kyushu University Fukuoka Japan

**Keywords:** crop damage, human–wildlife conflict, mast seeding, oak masting, red deer, resource pulses, wild boar

## Abstract

Human–wildlife conflicts, particularly the damage to agricultural crops caused by ungulates, pose significant ecological and economic challenges. Understanding the role of natural food availability in driving these conflicts is important for developing effective management strategies. We investigated how the pulsed availability of forest tree seeds, i.e., mast seeding, influences the extent of agricultural crop damage in Poland. Using a 19‐year national dataset (2001–2020), we analyzed the relationship between oak (*Quercus* spp.) and European beech ( *Fagus sylvatica* ) seed production, the abundance of wild boar ( *Sus scrofa* ) and red deer *Cervus elaphus* ), and the area of damaged agricultural crops. We found a negative relationship between oak seed production and the level of crop damage, with estimated damage decreasing by 30% from years of seed failure to years of abundant seed production, supporting the hypothesis that a diet shift occurs in ungulates during years of seed abundance that averts ungulates from damaging the crop. In contrast, beech seed production showed no significant effect on crop damage. Our findings demonstrate that pulsed resource dynamics in forests are an important driver of human–wildlife conflict in agricultural landscapes.

## Graphical abstract

Human–wildlife conflicts caused by ungulate damage to agricultural crops represent a major ecological and economic challenge. Using a 19‐year national dataset from Poland, we show that oak mast seeding substantially reduces crop damage—by approximately 30%—likely due to a dietary shift of ungulates toward forest resources, whereas beech seed production has no detectable effect. These findings highlight the importance of pulsed forest resources in shaping human–wildlife conflicts in agricultural landscapes.

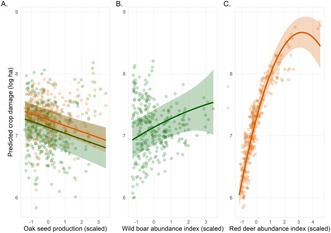

## 1 Introduction

Anthropogenic change has led many taxa to supplement natural foods with agricultural crops, including insects, birds, and especially ungulates (Bereś 2015; Vergin et al. 2025; Jacob et al. 2014; Blount et al. 2021; Montràs‐Janer et al. 2019; Brown et al. 2007). In wild boar, a synthesis of 11 studies across eight European countries showed frequent and often large consumption of crops with strong seasonal and geographic variation (Schley and Roper 2003). Crop damage is now a major form of human–wildlife conflict with ecological and socioeconomic costs. Habitat changes from natural to anthropogenic increase animal–human contact, and large‐bodied species commonly damage fields, livestock, orchards, and other property, fueling financial losses and negative attitudes (McKee et al. 2021; Hill 2018; Hulme et al. 2020; Distefano 2005; Basak et al. 2023). Because farmland provides accessible, energy‐rich food, wildlife turns to anthropogenic sources when natural resources are limited. For example, a 15‐year study in Italy reported fewer bear attacks on livestock in years of abundant beech masting but 67% higher attacks in poor mast years (Tattoni et al. 2025). These patterns show how fluctuations in natural food modulate the severity of conflict, highlighting the importance of identifying the ecological drivers of crop damage for better prediction and management. Financial compensation schemes have been introduced in many countries to support those affected (Ravenelle and Nyhus 2017), reaching tens of millions of euros annually (Coordination Rurale 2024; Bleier et al. 2012; Schley et al. 2008). Such losses burden farmers, institutions, and taxpayers, and mitigation strategies such as fencing, repellents, regulated hunting (Geisser and Reyer 2004), and supplementary feeding (Calenge et al. 2004) require further investment. Compensation records illustrate the scale of the problem: in 2000/2001 these amounted to nearly 26 million PLN in Poland (∼6 million EUR), rising to 104 million PLN (∼24 million EUR) in 2019/2020 (Główny Urzad Statystyczny 2006, 2021). Analysis of compensation records from the 2005/2006–2019/2020 hunting seasons showed that, on average, 1.72 thousand PLN was paid per hectare of damaged cropland nationwide (Figure S1 in Supporting Information). Pinpointing the environmental drivers of damage is thus important for both ecological understanding, forecasting, and cost‐effective management.

Ungulates are major contributors to crop damage due to their large body size, high energy demands, social foraging, and mobility. Wild boar ( *Sus scrofa* ) and red deer ( *Cervus elaphus* ) trample fields and consume large amounts of maize, cereals, potatoes, and rapeseed (Massei et al. 2015; Dzieciołowski 1979; Schley and Roper 2003; Picard et al. 1991). Behaviors such as grubbing alter soil structure (Mohr et al. 2005; Risch et al. 2010; Ribeiro et al. 2025), while group foraging increases the spatial extent of damage (Maselli et al. 2014). Red deer are herbivorous, shifting seasonally among grasses, shrubs, and conifer needles, and in autumn consume acorns and beechnuts (Gebert and Verheyden 2008; Barrere et al. 2020). Wild boar are omnivorous generalists but depend on energy‐dense resources such as acorns, turning to crops or alternative foods when mast is scarce (Massei et al. 2015). Thus, both species combine forest and agricultural foods, with mast availability shaping their autumn diets (Picard et al. 1991; Schley and Roper 2003).

Masting, the highly interannually variable and synchronized production of large seed crops, is a widespread strategy among European trees, including large‐seeded oaks (*Quercus* spp.) and European beech ( *Fagus sylvatica* ) (Kelly 1994; Bogdziewicz et al. 2024; Szymkowiak et al. 2024). By concentrating reproduction in high‐seeding years, trees enhance pollination and reduce seed predation (Zwolak et al. 2022). The resulting pulses of acorns and beechnuts provide critical resources for insects, birds, rodents, and ungulates (Thomas 2008; Myczko et al. 2014; Ruscoe et al. 2005; Schley and Roper 2003). While abundance of invertebrate seed predators is strongly regulated by mast cycles, large‐bodied vertebrates such as ungulates can compensate for poor seed years by shifting to alternative foods (Bogdziewicz et al. 2022; Curran and Leighton 2000). Nevertheless, fluctuations in seed abundance are expected to alter their foraging behavior, space use, the degree to which they exploit agricultural crops (Zwolak et al. 2022; Pucek et al. 1993), and to drive their population dynamics (Touzot et al. 2020; Gamelon et al. 2017).

Mast‐generated resource pulses also affect ungulate population dynamics. Wild boar depend on at least one energy‐rich food source throughout the year, whether mast or crops (Schley and Roper 2003). In high‐seeding years, acorn and beechnut availability improves body condition (Gamelon et al. 2017), advances reproduction (Drimaj et al. 2019; Cachelou et al. 2022), increases the proportion of breeding females (Gamelon et al. 2021), thereby promoting population growth (Massei et al. 1996; Touzot et al. 2020; Gamelon et al. 2021). Access to crops alongside mast can further enhance body size and condition (Merta et al. 2014). In contrast, mortality may increase when mast resources are unavailable, as observed in wild boar from Białowieża Forest, whereas red deer showed no such pattern (Okarma et al. 1995). In red deer, mast availability may not directly reduce mortality, but it can improve individual condition and reproductive investment (Peláez et al. 2018). Thus, while masting has particularly strong demographic effects on wild boar, it also shapes the feeding preferences of both species and modulates their reliance on agricultural foods.

Previous studies show that ungulates preferentially consume mast when available. In France, Picard et al. (1991) found that during oak high‐seeding year, acorns comprised 51% of red deer diet and occurred in 56% of rumen samples, while maize (12%), twigs (9%), and grasses (6%) were secondary. In the preceding poor seed production year, diets shifted toward grasses (20%), maize (12%), leaf stalks (13%), and beechnuts (9%). Long‐term data from La Petite Pierre Reserve in northeastern France support these patterns: over 30 years, both red and roe deer increased acorn intake in mast years, with red deer consuming up to 52% of diet compared to 34% in roe deer; in poor years, acorns dropped to 4% and 1% respectively (Barrere et al. 2020). Wild boar shows similar responses. A review by Schley and Roper (2003) highlights mast (e.g., seeds of oaks and beech) as a preferred food, with other items taken opportunistically. In the Czech Republic, stomach contents of 182 wild boars collected during a strong oak mast year showed acorns and maize dominating diets, with acorns reaching up to 95% of intake (Mikulka et al. 2018). As acorns declined in winter, maize consumption rose, and acorn presence disappeared. Together, these studies demonstrate that mast abundance strongly shapes ungulate diets. Yet most work has focused on dietary shifts, while the direct link between seed production and the extent of crop damage remains poorly quantified, despite its relevance for anticipating and mitigating human–wildlife conflict.

In this study, we tested whether oak and beech masting influences agricultural damage in Poland by altering ungulate foraging after seedfall. We predicted reduced crop damage in high‐seeding years and elevated damage when seed production failed. To examine this, we combined 19 years of national data (2001–2019) on oak and beech seed production, ungulate abundance, and reported crop damage. We aimed to clarify whether mast seeding modulates conflict intensity and to assess its potential value as an early‐warning indicator for targeted, cost‐effective management.

## 2 Materials and Methods

### 2.1 Research Area

Poland offers a suitable setting to study interactions between masting and crop damage due to its extensive coverage by both forests and farmland. Oaks ( *Quercus robur* , *Q. petraea* ) are widespread, while beech ( *Fagus sylvatica* ) reaches its northeastern range limit and is locally scarce (Eaton et al. 2016; Durrant et al. 2016). Forest cover increased modestly from 28.4% in 2001 to 29.5% in 2020 (Główny Urzad Statystyczny 2002, 2020), but connectivity remains low compared to other European countries (Estreguil et al. 2013). Agricultural land dominates the landscape, exceeding 50% of the national area, with sown fields declining from 12.3 to 10.7 million ha over the study period.

Ungulates are widespread across Poland (Borowik et al. 2013), occupying forests, farmland, and even urban areas (Kowalewska 2019). The main ungulate species are wild boar ( *Sus scrofa* ), red deer ( *Cervus elaphus* ), roe deer ( *Capreolus capreolus* ), and fallow deer ( *Dama dama* ), with moose ( *Alces alces* ) and bison ( *Bos bonasus* ) present regionally (Michalska et al. 2023; Mysterud et al. 2007). Wild boar populations increased for decades (Massei et al. 2015) but declined recently due to African Swine Fever (Szymańska and Dziwulaki 2022), whereas deer populations continue to expand across Europe (Burbaitė and Csányi 2010). Poland is divided into hunting districts managed by local associations, which also administer compensation for crop damage. For this study, we used voivodeship‐level (*N* = 16) population estimates reported by the Polish Hunting Association (PZŁ), which correspond to the highest‐level administrative divisions in Poland.

### 2.2 Seed Production Data

We extracted seed production data from MASTREE+, a data set that collects annual reproductive time series data from all over the world and makes these data freely available (Hacket‐Pain et al. 2022; Foest et al. 2024). We used data on European beech ( *Fagus sylvatica* ) and two types of oaks merged under one category ( *Quercus robur* and *Quercus petraea* , grouped together as *Quercus* spp.) from 2001 to 2019. This particular data set of seed production from Poland is a part of a long‐term monitoring program that began in 1951, covering 17 areas of the Regional Division of State Forests (RDLP). It consists of annual reports of estimates of the percentage of trees that fructified (to the nearest 10%) in a given year at each site, which captures the stand‐level extent of seed production (Kantorowicz 2000; Pesendorfer et al. 2020).

To spatially match the seed production data (collected at the level of RDLP) to voivodeships (the level at which crop damage and animal abundance data are collected), we transformed and weighted the seed production data to align with crop damage and animal abundance data, applying the following equation:

$$SP_{v,t}=\sum \left(SP_{f,t}*wt_{f}\right)$$

**FIGURE 1:**
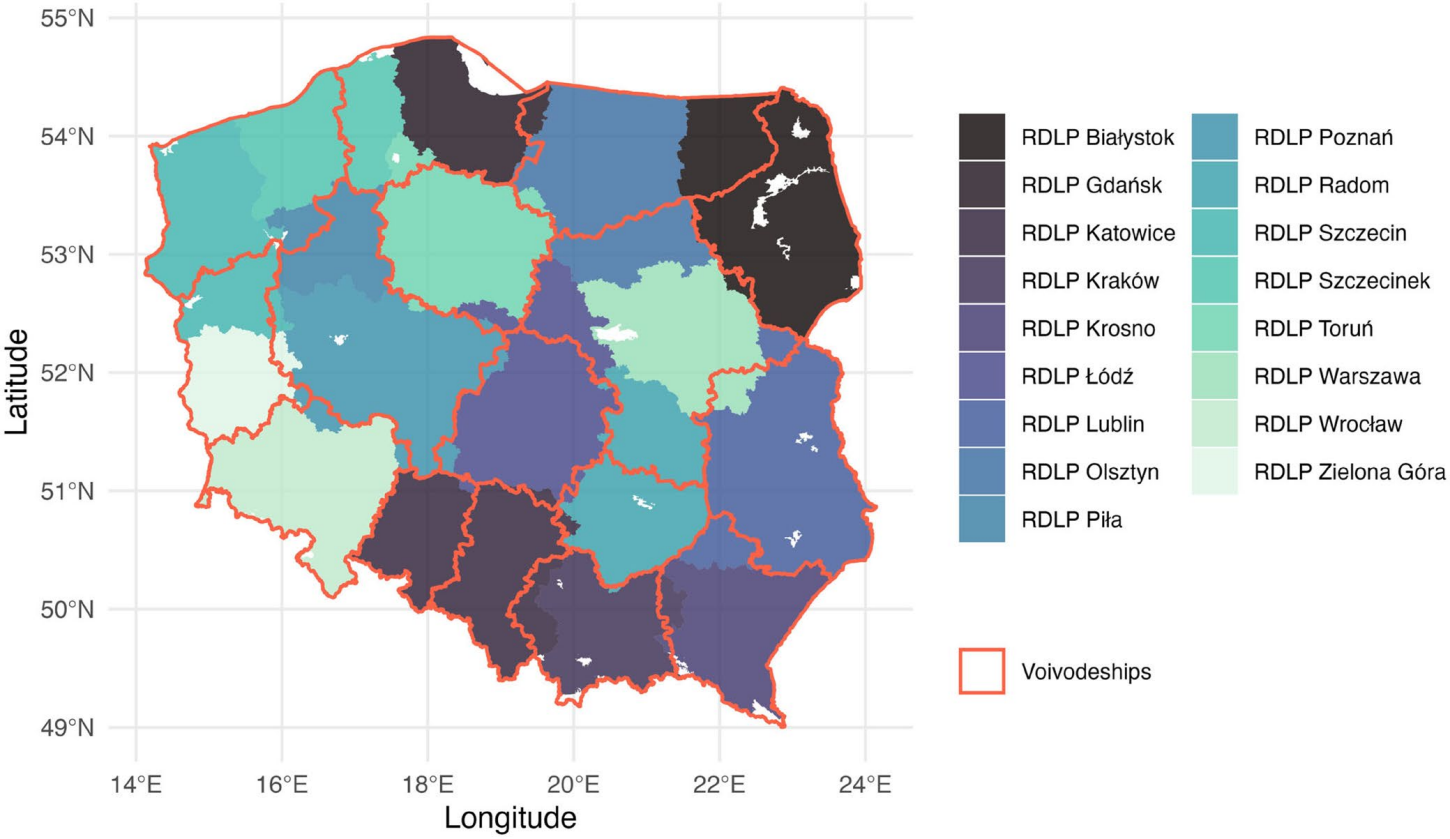
Map of the borders of Regional Directorates of State Forests (RDLP) and voivodeships (Open Forest Data 2020; Head Office of Geodesy and Cartography (GUGiK) 2026).

Seed production in a given voivodeship in a given year ($SP_{v,t}$) is calculated as a sum of seed production in each forest division within that year ($SP_{f,t}$), multiplied by the proportion of the forest division overlapping with the voivodeship ($wt_{f}$, map of divisions showed in Figure 1). The weighting procedure, however, had no impact on the qualitative outcome of our analysis.

**FIGURE 2:**
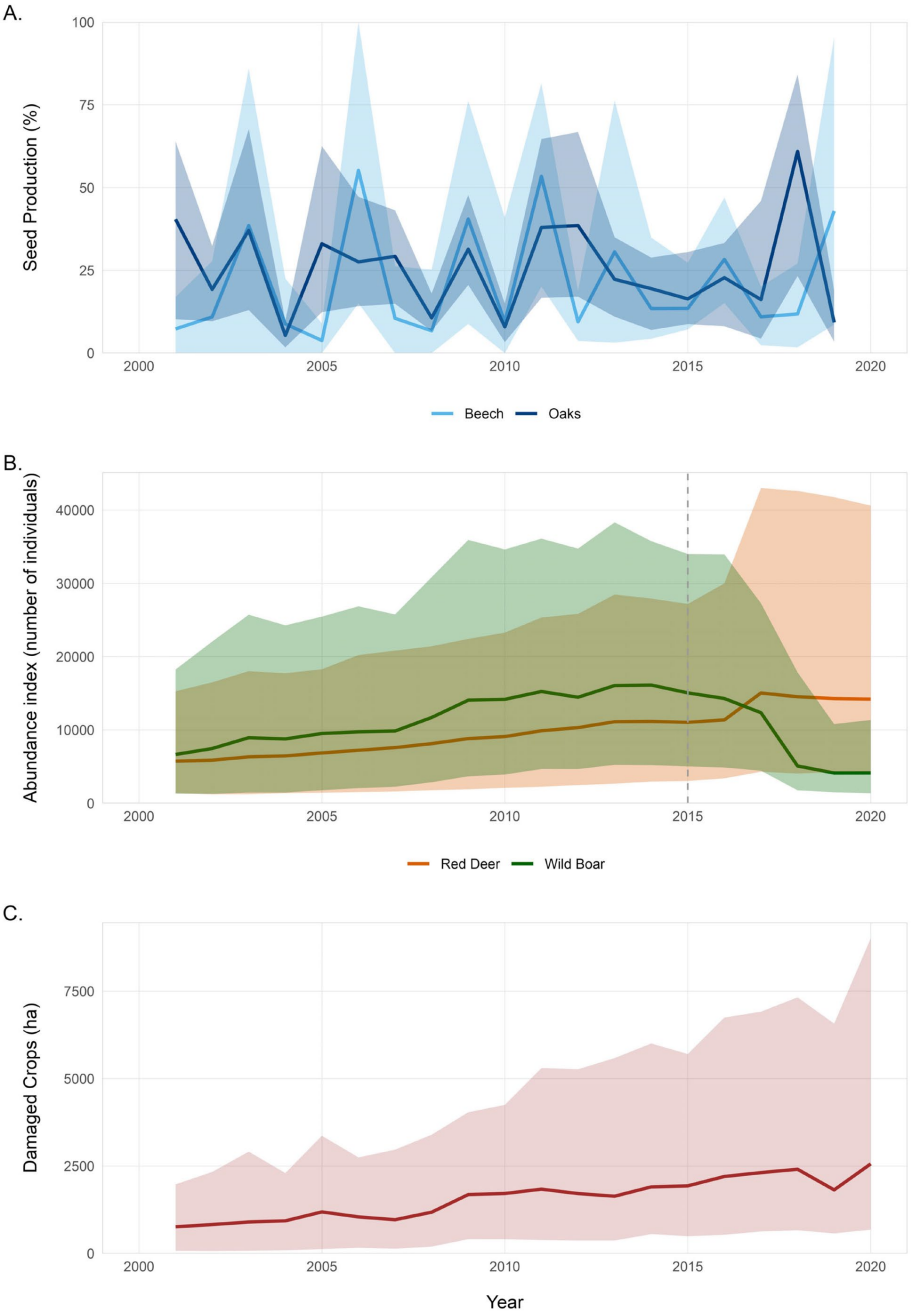
Temporal trends in seed production, ungulate populations, and crop damage across all study regions. (A) Annual seed production of beech and oaks over time. (B) Population trends of red deer and wild boar across all districts. (C) Annual crop damage area across all regions. Solid lines represent annual mean values, and shaded areas indicate the minimum–maximum range across study regions. The vertical dashed line indicates the African Swine Fever (ASF) outbreak (2015).

Seed production exhibited considerable interannual variation across years for both beech (mean coefficient of variation, CV = 1.02, and standard deviation, SD = 23) and oak (CV = 0.66, SD = 17) (Figure 2). We found a weak but statistically significant positive correlation between beech and oak seed production (*r* = 0.23, *p* < 0.001).

### 2.3 Damaged Crops and Ungulate Abundance Proxy

We obtained data on reduced crop damage area (hectares) and ungulates populations (number of individuals) spanning 19 hunting seasons from 2000/2001 to 2019/2020 at the voivodeship level, from the Research Station of the Polish Hunting Association (PZŁ) in Czempiń. Their main objective since 1990 has been to monitor game species in Poland. Damaged crop data is a set of annual records of reduced area of damaged agricultural crops for each voivodeship, calculated as the damaged crop area multiplied by the percentage of its destruction. Damaged crop areas and compensation withdrawals for these damages are documented by the divisions of PZŁ and aggregated in annual reports. Crop damages are reported by farmers and subsequently assessed by hunting district lessees or managers. Reported damage must be declared in a specific and timely manner and reach a minimum threshold. PZŁ is responsible for the majority of hunting compensations in Poland (∼73% in 2000/2001, and ∼88% in 2019/2020 hunting season, according to Główny Urzad Statystyczny 2002, 2020).

As a proxy for ungulate abundance, we used the number of individuals for each ungulate species estimated in April each year for each forest district, then grouped by RDLP regions and voivodeships. This data is used to create annual hunting plans for the season ahead (therefore prone to bias). Estimates are made by representatives of hunting districts and foresters which are working daily in the field based on direct observations of animals, knowledge of crop and forest damage caused by ungulates, and information from hunting activities. This method is an official assessment to provide inventory by the government and represents trends in populations of animals. It is thought to be a valuable tool to estimate population sizes (Hušek et al. 2021), especially for big animals moving in permanent groups. Additionally, sporadically in some regions other methods are used as supplementary such as counting animals with drones or drive counts. We focused on two ungulate species that are widespread and known for damaging crops and consuming beech nuts and acorns: wild boars ( *Sus scrofa* ) and red deer ( *Cervus elaphus* ).

Crop damage area showed an increasing trend, rising from under 1000 ha in 2001 to approximately 2500 ha in 2020 and was also characterized by relatively large interannual variation (CV = 0.77, SD = 1248, Figure 2). Regarding animal populations, ungulate numbers generally increased over the studied period; however, after 2014, the wild boar population declined sharply due to African Swine Fever (ASF) (Figure 2).

### 2.4 Statistical Analysis

We modeled the relationship between crop damage and seed production, taking into account ungulate abundance. We used a generalized linear mixed model with reduced damaged crop area as a response variable, and predictors including two ungulate species abundance (wild boar and red deer, separately) and seed production from two species (beech and oak, separately). All predictors were scaled to enable direct comparisons between them. After visual inspection of the relationships, animal abundance variables were fitted with quadratic terms to allow for nonlinear relationships. We log‐transformed the crop damage records to address skewness in the data (see Figure S2 in Supporting Information). The region (voivodeship) was included as a random intercept to account for spatial variations in crop damage and repeated sampling. We also tested the interaction between ungulate abundance and seed production. However, it was nonsignificant and was therefore excluded from the model (Table S1 in Supporting Information).

We lagged seed production by 1 year to match the timing of the hunting season (Figure S3 in Supporting Information), which runs from April 1st to March 31st of the following year. The reduced damage area data is referenced to the hunting season (e.g., the 2000/2001 season is recorded as 2001), while the ungulate counts represent the estimated population at the end of March, which is then used to create the annual hunting plans for the upcoming season. Seed production is estimated in a given year and reflects the food availability for ungulates after the seedfall (within the hunting season, e.g., seeds produced in 2000 are a food source in 2000/2001 hunting season).

To assess the potential impact of African Swine Fever (ASF) on crop damage, we additionally ran models on a shortened time span, using 2015 as a cutoff year (responding to the 2014/2015 hunting season, within which the first known ASF outbreak happened in Poland). Due to insufficient post‐2015 data and the progressive nature of the disease, a classical before–after analysis was not feasible. Therefore, we limited the analysis to the pre‐outbreak period and compared it with results from the full dataset.

To avoid issues of multicollinearity between the two ungulate species, which were highly correlated (*r* = 0.68 for the full period; *r* = 0.91 before ASF), we fitted separate models for wild boar and red deer.

All analyses were conducted in R (version 4.4.2). We fitted the models via the lme4 package (version 1.1.35.5) (Bates et al. 2015). To generate model‐based predictions, we used the ggeffects package (Lüdecke 2018).

## 3 Results

Oak seed production was negatively related to agricultural crop damage, while beech seed production showed no significant effect (p>0.1).

**FIGURE 3:**
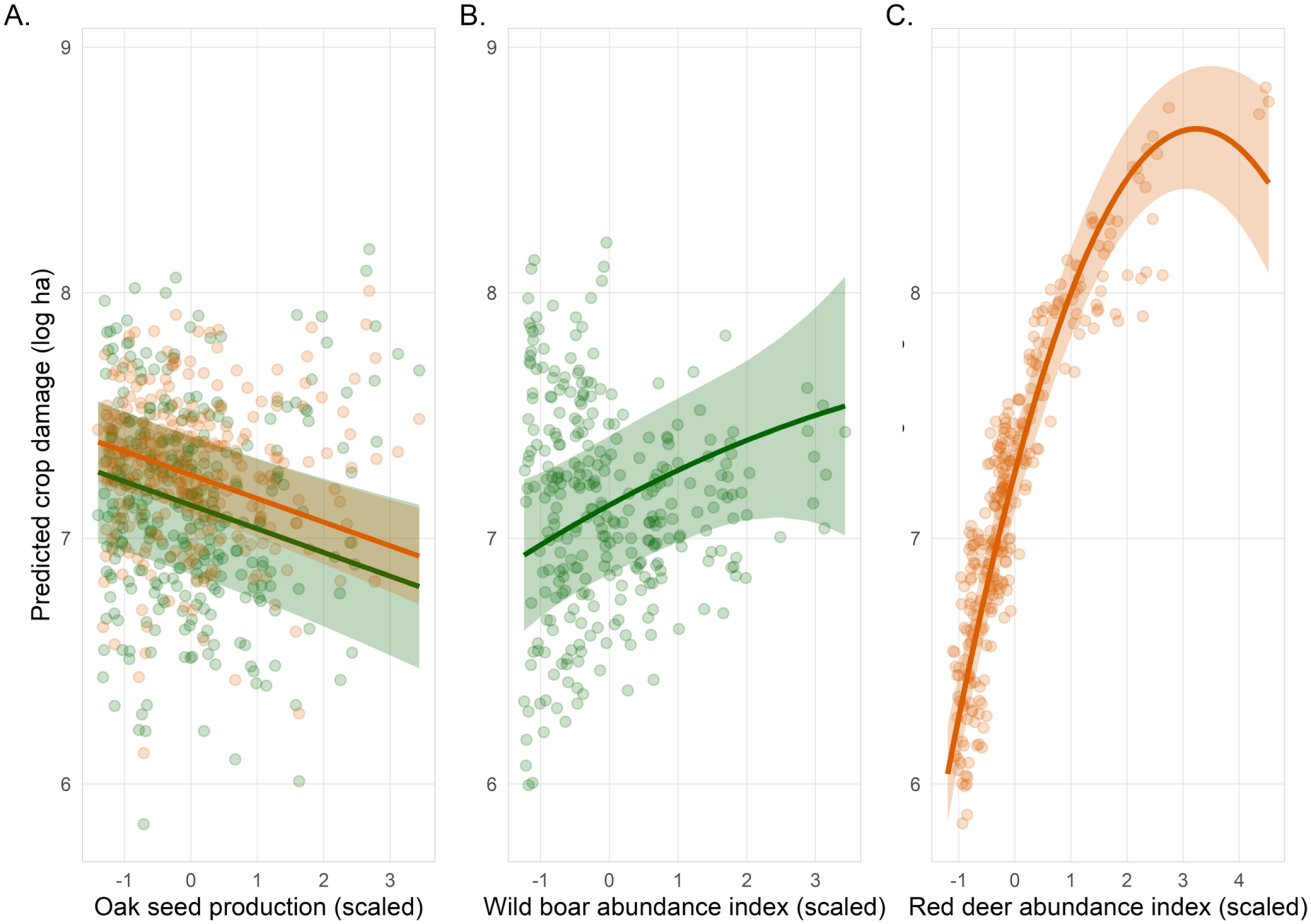
Estimated relationships between crop damage and ecological predictors across the full study period. Panels show estimated crop damage (ha) in relation to (A) oak seed production, (B) wild boar abundance, and (C) red deer abundance. Beech seed production was not a significant predictor of crop damage (Table S2 in Supporting Information). Lines represent estimated mean values with 95% confidence intervals (shaded areas), and points show partial residuals. Colors show predictions derived from separate models (green—that included wild boar abundance as a predictor; orange—red deer abundance). Predictors were scaled before entering the model. Estimates were derived from linear mixed models with voivodeship included as a random intercept.

In the model including wild boar abundance, oak masting significantly reduced crop damage (β=−0.096, SE=0.025, p<0.01), while wild boar abundance had a strong positive effect ($\beta_{1}=0.151$, SE=0.049, p=0.002, $\beta_{2}=-0.01$, SE=0.024, p=0.689). Assuming average conditions, predicted damage decreased from ∼1422 ha at minimal oak seed production (1.75%) to ∼892 ha at maximum acorn production (84.19%, Figure 3A), representing a ∼37% reduction in actual area damaged. In contrast, wild boar abundance had a strong positive effect, where predicted crop damage increased from ∼1024 ha at minimal abundance (1278 individuals) to ∼1880 ha (Figure 3B) at highest abundance (38,329 individuals), corresponding to more than a 80% increase.

In the red deer model, oak seed production again had a negative effect (β=−0.096, SE=0.017, p<0.001), where assuming average conditions predicted crop damage decreased from ∼1422 ha at minimal oak seed production (1.75%) to ∼893 ha, which accounted for a ∼37% reduction. Red deer abundance was a strong positive predictor of crop damage ($\beta_{1}=0.864$, SE=0.047, p<0.001, $\beta_{2}=-0.133$, SE=0.013, p<0.001). Predicted damage increased from ∼415 ha at minimal abundance (1221 individuals) to ∼4656 ha at the highest values (43,007 individuals)—a more than tenfold rise (Figure 3C).

Across both model configurations, oak masting reduced crop damage, while ungulate abundance remained the dominant positive driver. The negative effects of oak masting on crop damage persisted when analyzing only the period before the ASF outbreak (Table S2 in Supporting Information).

## 4 Discussion

Our results show that interannual variation in seed production can impact ungulate crop utilization. We found a negative relationship between acorn availability and the area of damaged crops, with predicted damage decreasing by over 30% between years of lowest and highest oak seed production. This indicates that high‐seeding years buffer conflict by reducing the need for ungulates to forage in fields. The pattern suggests that energy‐rich acorns act as a resource, lowering reliance on agricultural foods when abundant, while poor mast years intensify pressure on farmland. By linking dietary flexibility of ungulates to a direct, management‐relevant outcome, these results extend earlier work that focused mainly on diet composition. The correlation between oak seed production and crop damage suggests a dietary shift. However, other mechanisms, such as changes in population dynamics and ungulate movement in response to masting in subsequent years, may be involved. Practically, while animal abundance is the key determinant of crop damage, acorn availability emerges as a useful indicator of conflict risk: integrating mast seeding with regional factors such as forest composition, proximity of farmland to oak stands, and animal movements across administrative borders could support more targeted deployment of mitigation strategies.

In contrast to oak, beech seed production did not show a significant influence on crop damage in our analyses. This could be due to the lower nutritional value and smaller size of beechnuts compared to acorns (Rivero et al. 2019). A study by Gamelon et al. (2017) showed that both acorn and beech mast have an impact on wild boar populations, through direct and indirect effects. It also showed that population dynamics and fertility can depend on both the same year and last year's seed production, as well as ongoing hunting pressure. Here, however, at a country scale, working on estimates not specific to population, we could not test lagged effects on ungulate abundance, and with no data on hunting practices, we could not include that as a factor. Another issue could be the distribution of the beech forest in Poland, as it is not as common as oaks, and therefore, beech seed availability may be smaller. From a management perspective, our results imply that monitoring oak flowering intensity in Poland can be a valuable forecasting tool, whereas tracking beech flowering may be less critical on a country‐level, given its negligible impact, but we acknowledge it may be important in some regions.

Our analysis indicates that the abundance of key game species had a substantial impact on the extent of crop damage, and it has been shown in previous studies in Poland that agricultural crops are a significant component of their diet (Gebert and Verheyden 2008; Kniżewska and Rekiel 2015). We observed that recent (since 2015), drastic shifts in the ungulate community structure, i.e., rising deer numbers and the decline of wild boar due to African Swine Fever (ASF), did not erase the overall significant influence of oak seed production. It is, however, important to note that the wild boar population decline was driven by several interconnected factors: direct mortality from the disease, extensive sanitary culling, and intensified hunting pressure related to management policies, with spatially variable intensity across regions. The subsequent spatiotemporal spread of the epidemic (Wojewódzki Inspektorat Weterynarii 2019) likely had profound and regionally varied effects. However, the limited number of observations in the post‐ASF period prevented us from conducting a formal statistical comparison between the two periods.

It is important to acknowledge, however, that our analysis of animal abundance proxies is based on hunter observations and therefore subject to methodological limitations, as observer expertise and financial incentives can introduce biases into population estimates (Kamieniarz et al. 2023). This data is produced to guide management actions, not to reflect true population sizes. These limitations prevented us from assessing a direct numerical response of ungulates to seed production, but it remains evident that animal abundance is a critical factor intensifying human–wildlife conflicts. Additionally, the strong correlation between wild boar and red deer abundances proxies, including within sites, prevented reliable disentanglement of their independent effects and their combined contribution to crop damage.

The species analyzed are game animals, and hunting remains the primary population management tool in Europe. However, high hunting pressure can induce fear, altering animal behavior—potentially creating “refugee effects” (Amici et al. 2012) where animals avoid certain areas—and influencing their use of agricultural landscapes (Sütő et al. 2023). Hunting pressure can also drive evolutionary consequences, such as earlier reproduction in wild boars (Gamelon et al. 2011). Furthermore, hunting pressure might obscure the effects of seed production on population dynamics at local scales, for example, by maintaining animal populations at low levels. Management recommendations should therefore integrate these environmental factors to better forecast potential crop losses. While the ongoing recovery of large predators like wolves in Poland (Chapron et al. 2014; Di Bernardi et al. 2025) could theoretically help regulate ungulate populations, habitat fragmentation and other anthropogenic influences may limit their effectiveness in keeping ungulate numbers under control (van Beeck Calkoen et al. 2023). Therefore, it could be beneficial to align hunting strategies with oak seed production, as masting can influence not only the extent of wildlife impact on agriculture, but also population dynamics in the subsequent months (Gamelon et al. 2017; Touzot et al. 2020).

The limitation of this dataset is a lack of landscape factors, which could explain the dynamics further. However, the fact that we detected this effect using broad‐scale, nontargeted data suggests that in oak‐rich landscapes with strong fruiting, the local strength of this relationship may be even greater. Another limitation relates to crop damage data. This data is restricted to the Polish Hunting Association (PZŁ), as it is the main payer of hunting compensation in Poland. Compensation for damage to croplands located outside hunting districts is covered by the state budget, the State Forests National Forest Holding (Lasy Państwowe), or, in the past, the Agricultural Property Agency. We did not include damages outside hunting districts in our study, as only annual compensation totals were publicly available. Crop damage within PZŁ is declared by farmers. The reporting process is time‐sensitive, requiring notification within a few days, followed by assessment by professionals. This may lead to underreporting, particularly for small damages or missed deadlines. Finally, changes in legislation and compensation schemes over time may have influenced both reporting behavior and damage valuation, introducing additional sources of bias.

In conclusion, our findings demonstrate that oak seed production plays a significant role in shaping crop damage by altering ungulate foraging behavior. This interaction occurs within a complex socio‐ecological context influenced by climate, habitat structure, and human management, suggesting that integrating regional seed production forecasts with landscape composition data and adaptive wildlife management practices may provide substantial benefits for mitigating human–wildlife conflicts. Future research should adopt a regional‐scale perspective and incorporate detailed data on landscape characteristics, detailed ungulate abundance data, stomach contents, and seed production for both species to improve understanding of the observed patterns.

## Author Contributions

**Maria A. Bogdańska:** conceptualization (equal), formal analysis (equal), visualization (equal), writing – original draft (equal). **Michał Bogdziewicz:** conceptualization (equal), writing – review and editing (equal). **Valentin Journé:** conceptualization (equal), formal analysis (equal), visualization (equal).

## Funding

This work was supported by the European Research Council, 101039066.

## Conflicts of Interest

The authors declare no conflicts of interest.

## Supporting information


**Table S1:** Interaction effects between seed production and ungulate abundance on crop damage. The retained model summaries are provided in Table S2.
**Table S2:** Linear Mixed‐Effects Regression fixed effects for seed production and ungulate abundance for full period and before the ASF outbreak. Animal abundance variables were modeled using quadratic terms to capture potential nonlinear relationships.
**Figure S1:** Mean compensations per 1 ha of damaged crop area for all regions in hunting seasons 2005/2006–2019/2020. The red line represents the calculated mean for the whole of Poland.
**Figure S2:** Distribution of damaged crop area (ha) before (A) and after (B) log‐transformation.
**Figure S3:** Timeline of the variables used in the study. Crop damage is reported in the spring of the year T, and covers the 12‐month period before the report. Ungulate abundance proxy is reported in the spring of the year T, and thus covers the same period as damage; we left it unlagged. Seed production in year T happens in autumn, which necessitates lagging (T‐1) by 1 year.

## Data Availability

The data and code supporting the results will be archived in the Open Science Framework (OSF): https://osf.io/q5zak/overview?view_only=f9f8ad986fe14bc68999ce05d7a5d49a.
