# Supplementary material for: Acorn Availability Reduces Agricultural Damage by Ungulates
Source: Ecol Evol. 2026 Jul 27;16(7):e73974. doi: 10.1002/ece3.73974 (PMC13403050; doi:10.1002/ece3.73974)
Supplement: Supplementary file 1 — Table S1: Interaction effects between seed production and ungulate abundance on crop damage. The retained model summaries are provided in Table S2. Table S2: Linear Mixed‐Effects Regression fixed effects for seed production and ungulate abundance for full period and before the ASF outbreak. Animal abundance variables were modeled using quadratic terms to capture potential nonlinear relationships. Figure S1: Mean compensations per 1 ha of damaged crop area for all regions in hunting seasons 2005/2006–2019/2020. The red line represents the calculated mean for the whole of Poland. Figure S2: Distribution of damaged crop area (ha) before (A) and after (B) log‐transformation. Figure S3: Timeline of the variables used in the study. Crop damage is reported in the spring of the year T, and covers the 12‐month period before the report. Ungulate abundance proxy is reported in the spring of the year T, and thus covers the same period as damage; we left it unlagged. Seed production in year T happens in autumn, which necessitates lagging (T‐1) by 1 year. [file ECE3-16-e73974-s001.pdf]

# Supporting Information

Table S1: Interaction effects between seed production and ungulate abundance on crop damage. The retained model summaries are provided in Table S2.

| Interaction | | Estimate | Std. E. | p.value |
|---|---|---|---|---|
| Wild boar abundance | : Beech seed production | 0.009 | 0.026 | 0.72 |
| Wild boar abundance | : Oak seed production | -0.005 | 0.029 | 0.86 |
| Red deer abundance | : Beech seed production | 0.014 | 0.015 | 0.34 |
| Red deer abundance | : Oak seed production | 0.005 | 0.017 | 0.79 |

Table S2: Linear Mixed-Effects Regression fixed effects for seed production and ungulate abundance for full period and before the ASF outbreak. Animal abundance variables were modeled using quadratic terms to capture potential non-linear relationships.

| Model | Predictor | Estimate | Std. E. | df | p.value |
|---|---|---|---|---|---|
| Wild boar full period | Beech seed production | 0.035 | 0.025 | 283 | 0.156 |
| | Oak seed production | -0.096 | 0.026 | 284 | <0.001 |
| | Wild boar abundance | 0.152 | 0.049 | 299 | 0.002 |
| | Wild boar abundance$^2$ | -0.010 | 0.025 | 293 | 0.689 |
| Red deer full period | Beech seed production | 0.008 | 0.017 | 285 | 0.659 |
| | Oak seed production | -0.096 | 0.017 | 286 | <0.001 |
| | Red deer abundance | 0.864 | 0.047 | 255 | <0.001 |
| | Red deer abundance$^2$ | -0.133 | 0.013 | 298 | <0.001 |
| Wild boar before ASF | Beech seed production | 0.012 | 0.016 | 206 | 0.430 |
| | Oak seed production | -0.059 | 0.016 | 207 | <0.001 |
| | Wild boar abundance | 0.845 | 0.038 | 199 | <0.001 |
| | Wild boar abundance$^2$ | -0.156 | 0.018 | 219 | <0.001 |
| Red deer before ASF | Beech seed production | 0.017 | 0.019 | 205 | 0.380 |
| | Oak seed production | -0.089 | 0.019 | 206 | <0.001 |
| | Red deer abundance | 0.863 | 0.055 | 176 | <0.001 |
| | Red deer abundance$^2$ | -0.137 | 0.021 | 219 | <0.001 |

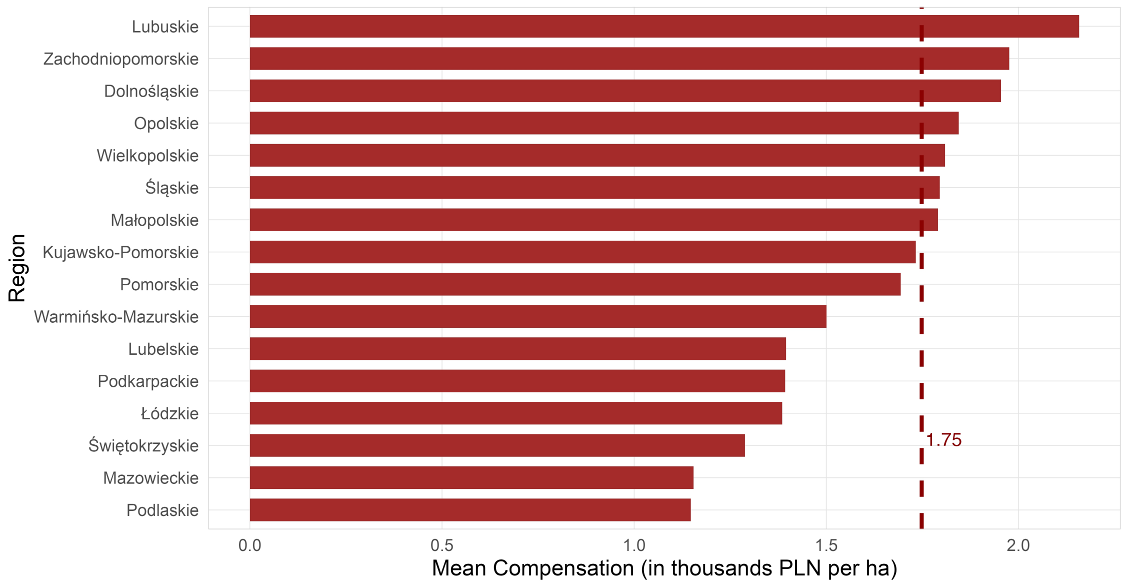


Figure S1: Mean compensations per 1 hectare of damaged crop area for all regions in hunting seasons 2005/2006–2019/2020. The red line represents the calculated mean for the whole of Poland.

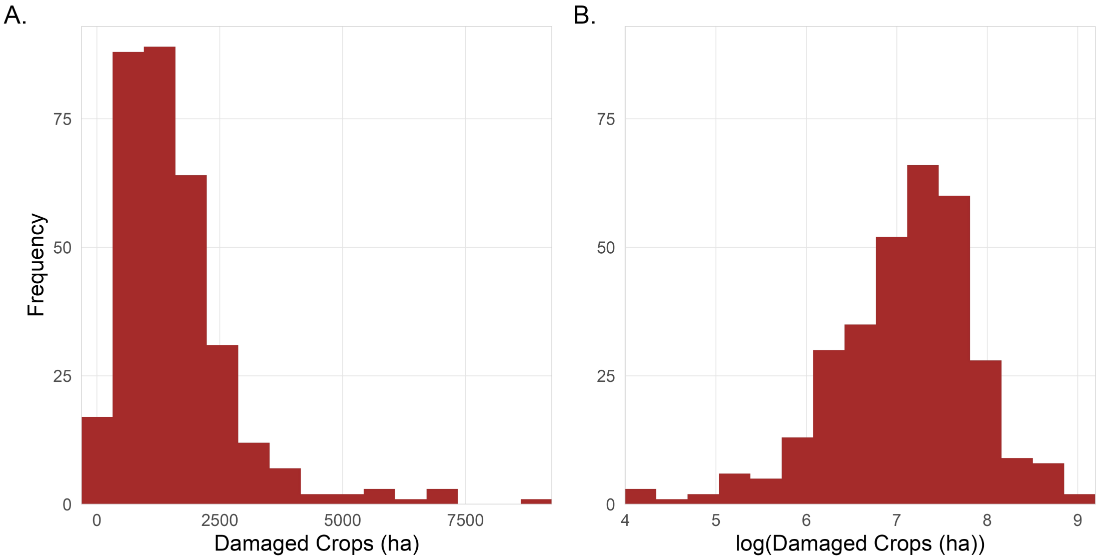


Figure S2: Distribution of damaged crop area (ha) before (A.) and after (B.) log-transformation.

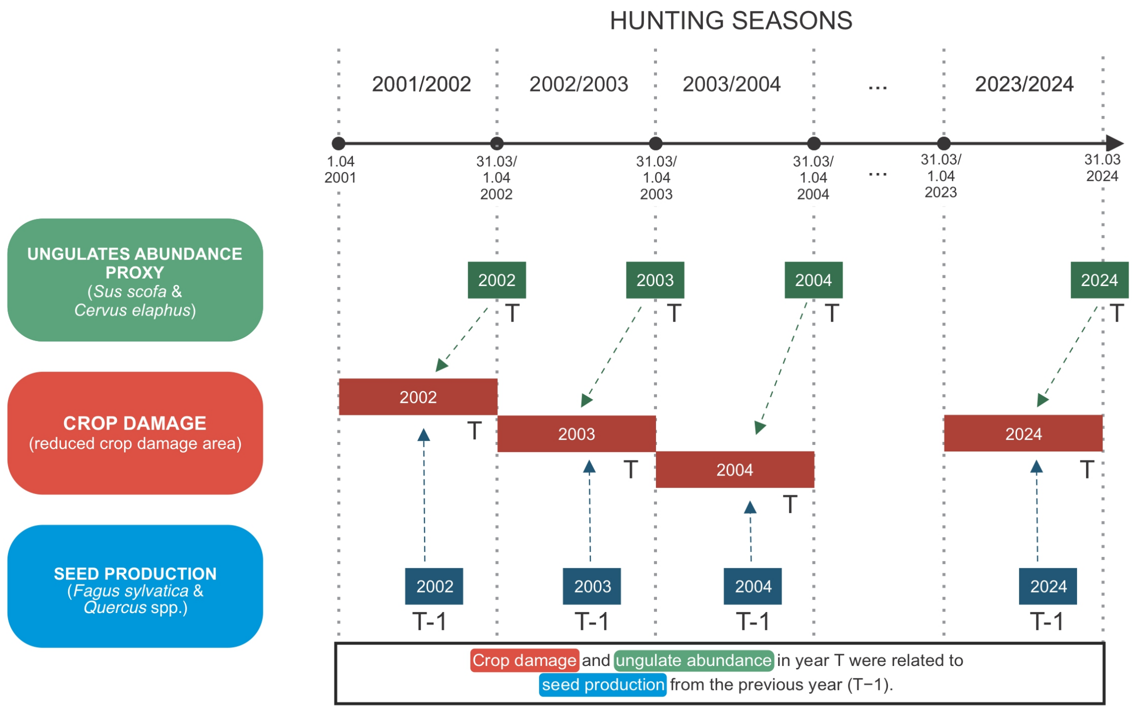


Figure S3: Timeline of the variables used in the study. Crop damage is reported in the spring of the year T, and covers the 12-month period before the report. Ungulate abundance proxy is reported in the spring of the year T, and thus covers the same period as damage; we left it unlagged. Seed production in year T happens in autumn, which necessitates lagging (T-1) by one year.
